# Identification, Biology, and Bactericide Control of Peach Bacterial Shot Hole in Hebei Province, China

**DOI:** 10.3390/microorganisms14061179

**Published:** 2026-05-23

**Authors:** Jianchao Cui, Haijiao Xu, Liying Fan, Yu Wang, Limin He, Zhaoyuan Wang, Jicheng Han, Jie Li, Qihang Tian, Wenshi Zhao, Yonghong Li

**Affiliations:** Changli Institute of Pomology, Hebei Academy of Agriculture and Forestry Sciences, Qinhuangdao 066600, China; cjc19880320@126.com (J.C.); xuhaijiao1234@sina.cn (H.X.); fanliying123456@163.com (L.F.); wy912963518@163.com (Y.W.); helimin122@163.com (L.H.); wangzhaoyuan1981@sina.com (Z.W.); hanjicheng6902@126.com (J.H.); lijie-19850908@126.com (J.L.); mstianqihang@126.com (Q.T.); zws1325855900@163.com (W.Z.)

**Keywords:** peach, bacterial shot hole, *Xanthomonas arboricola* pv. *pruni*, biological characteristics, bactericide screening

## Abstract

Peach bacterial shot hole is a major disease limiting the yield and quality in most peach-producing areas worldwide. To clarify its etiology and support the development of targeted management strategies, diseased samples were collected from Changli County peach orchards. The pathogen was isolated, purified and verified by Koch’s postulates. Based on morphological, biochemical and multi-locus phylogenetic analyses, the causal agent was identified as *Xanthomonas arboricola* pv. *pruni* (isolate TCK-5). Biological characterization revealed that TCK-5 grew optimally in KB and NB medium at 28 °C, pH 7.0–7.5 and 0.5–1.0% NaCl, efficiently utilized glycerol and organic nitrogen source (proteose peptone, beef extract and yeast extract), with light showing no significant effect on growth. The strain TCK-5 exhibited a lethal temperature of 51 °C, indicating that heat treatment above this threshold effectively disinfects pruning tools and contaminated plant debris. Among 18 bactericides tested in vitro, biological bactericide outperformed chemical ones, with 0.3% Tetramycin AS (EC_50_ = 0.1051 mg/L) and 3% Zhongshengmycin SL (EC_50_ = 2.9252 mg/L) exhibiting the strongest inhibitory activity. This study fills a regional knowledge gap in the epidemiological distribution of the pathogen in northern China and advances current understanding of *X. arboricola* pv. *pruni* occurrence, providing a scientific basis for subsequent epidemic monitoring and integrated control of peach bacterial shot hole.

## 1. Introduction

Peach (*Prunus persica* L.) is an economically important stone fruit crop in China, with a production of 17.6168 million tons valued at over US $13.3194 billion (FAOSTAT, http://faostat.fao.org, accessed on 6 May 2026). However, driven by extreme weather variability, changes in orchard management, and cultivar replacement, peach bacterial shot hole is now reported in 44 countries around the world (prevalent across Asia, Europe, North America and Oceania), causing widespread epidemic outbreaks and continuous economic losses [1]. For example, fruit disease incidence reached 25–75% in the United States, 30% in northern Italy, and 5.4–75.9% in a 12-year survey in Okayama Japan [2]. Moreover, a severe outbreak with a disease incidence of 18–67% has occurred in several orchards of Hebei Province, the major producing region in northern China [3]. The disease affects leaves, twigs, and fruits, causing progressive tree decline and significant economic losses [2,4].

Several bacterial pathogens have been reported to cause shot hole symptoms in peach, primarily including *Xanthomonas arboricola* pv. *pruni* [5], *Pantoea agglomerans*, *Pantoea ananatis* [6], *Bacillus cereus*, *Bacillus thuringiensis* and *Bacillus pacificus* [4]. Nevertheless, the symptoms induced by these different genera are relatively similar [4,6], making it difficult to distinguish the causal agent based on symptomatology alone. Moreover, pathogen types and community structure are strongly influenced by geographical location. For instance, *Xanthomonas* spp. were predominantly isolated from regions of provinces in China such as Hubei, Shandong and Xizang, while only *Pantoea* spp. were obtained from Guangdong, Guangxi, and others, with no pathogenic *Xanthomonas* detected [7]. However, systematic etiological studies of this disease in Hebei Province are still scarce, resulting in a limited understanding of its pathogen taxonomy and key biological traits, which hinders the design of precise and locally adapted control strategies.

Precise pathogen identification is fundamental to effective disease control. Traditional pathogen identification based on morphological, physiological, and biochemical characteristics lacks the resolution to distinguish closely related species and pathovars [8]. In recent years, multi-locus sequence analysis (e.g., 16S rRNA, *gyrB*, *dnaK*, *efP*, and *atpD*) and whole-genome sequencing have emerged as powerful tools for classification and identification of pathogenic bacteria [9]. These housekeeping and ribosomal genes are widely accepted as standard molecular markers for *Xanthomonas* species identification and phylogenetic analysis, as each possesses sufficient genetic variation and taxonomic resolution to distinguish closely related strains at the species and subspecies levels [7]. Compared with whole-genome sequencing, multi-locus sequence analysis based on these markers is more cost-effective and time-saving, making it suitable for large-scale sample detection and phylogenetic clustering without compromising taxonomic reliability. In addition, disease control in current production still relies on chemical bactericides for emergency intervention, due to their rapid efficacy and ease of application [10]. However, previous studies have shown that long-term irrational use of chemicals has led to inconsistent control efficacy. For instance, the use of copper-based compounds (copper hydroxide) and agricultural streptomycin has been reported to select for resistance in *Xanthomonas* spp. from tomato [11] and peach [12]. In addition, the resulting pesticide residues threaten fruit quality, safety, and the ecological environment [13]. Therefore, screening for highly effective, low-toxicity, and low-residue bactericides helps to guide scientific field application, delay resistance development, and promote sustainable peach production [10].

To enable accurate prevention and control of bacterial shot hole, this study focused on isolating and purifying the pathogen, assessing its pathogenicity, and conducting morphological and molecular identification on samples collected from peach orchards in Hebei Province. Additionally, to further understand the pathogen’s environmental adaptability, this study investigated its key biological traits (e.g., preferences for light, temperature, pH, and nutrients) and screened potential bactericides in vitro to identify effective candidates. This research aims to provide a theoretical and technical basis for the early diagnosis and control of peach bacterial shot hole, thereby filling the research gap regarding its causal pathogen in northern China; while also providing a scientific basis for the accurate diagnosis and targeted regional management of this disease, ultimately contributing to efficient, stable, and sustainable peach production.

## 2. Materials and Methods

### 2.1. Sample Collection and Pathogen Isolation

In June 2024, leaf samples of peach (*P. persica*) exhibiting typical symptoms of bacterial shot hole were collected from two commercial orchards in Changli County, Hebei Province, China (119°13′12″ E, 39°46′69″ N; 118°5′25″ E, 39°53′18″ N). In each orchard, 5 trees were randomly selected following a diagonal five-point sampling pattern, and 3–5 symptomatic leaves were collected from each tree. All samples were placed in sterile plastic bags and transported to the laboratory for further processing.

Bacteria were isolated following Zang et al. with minor modifications [6]. The symptomatic tissues were washed with sterilized water, dried, and cut into 5 × 5 mm small pieces. Surface sterilization was performed by sequential immersion in 2% (*w*/*v*) sodium hypochlorite (Lingfeng Chemical Reagent Co.,Ltd., Shanghai, China) for 30 s, 75% (*v*/*v*) ethanol (Oubokai Chemical Reagent Co., Ltd., Tianjin, China) for 2 min, and sterile distilled water for rinsing three times. Ten sterilized pieces from different leaves were ground in 1 mL of sterile distilled water, and the resulting suspension was then plated onto NA (Table S3) plates and incubated at 28 °C for 48 h. Single colony was inoculated into fresh NB (Table S3) broth (three single colonies per isolate), followed by incubation in a shaker (MQD-S2AR, Minquan Co., Ltd., Shanghai, China) at 28 °C with shaking at 180 rpm for 24 h. An equal volume of 80% (*v*/*v*) glycerol (Kaitong Chemical Reagent Co., Ltd., Tianjin, China) was mixed with the bacterial culture, and stored at −80 °C.

### 2.2. Pathogenicity Test

For pathogenicity assays [6], healthy leaves and fruits of field-grown 8-year-old peach (cv. Zhongtaohongyu) were surface-sterilized with 75% ethanol, thoroughly rinsed with sterile water, and air-dried. Bacterial suspension was prepared by culturing single colony in NB broth at 28 °C with shaking at 180 rpm for 24 h, then centrifuged and resuspended in sterile distilled water to adjust the concentration to an OD_600_ of 0.05 using a spectrophotometer (TU-1950, Puxi Co., Ltd., Beijing, China). The bacterial suspension (0.05 OD_600_) was applied using a hand-held spray bottle, sprayed evenly onto both surfaces of leaves and onto the entire surface of fruits until run-off (approximately 1 mL per leaf and 2 mL per fruit). Sterile distilled water was used as a negative control. All inoculated tissues were covered with plastic bags for 48 h to maintain high humidity. The experiment was performed with two independent biological replicates with three technical replicates per treatment (20 leaves and 6 fruits each). Pathogenicity was assessed qualitatively by observing symptom development every 2 days for 2 weeks. To complete Koch’s postulates, the pathogen was reisolated and identified from symptomatic tissues.

### 2.3. Morphological Identification and Biochemical Assay

Morphological characteristics of the isolates were examined, including colony morphology (e.g., color, shape, and texture) on NA medium. Micromorphological features were observed using an Olympus BX53 optical microscope equipped with an Olympus DP74 color camera (Olympus, Tokyo, Japan), 200 cells were randomly measured.

Gram staining was performed using a Weigert’s Gram Stain Kit G3230 (Beijing Solarbio Science & Technology Co., Ltd., Beijing, China) according to the manufacturer’s instructions. Biochemical characteristics of the bacterium were performed according to Balaž et al. [14], including assays of oxidase, catalase and starch hydrolase activities, hydrogen sulfide (H_2_S) production, gelatin liquefaction, esculin hydrolysis, nitrate reduction, and citrate utilization.

### 2.4. PCR Amplification and Phylogenetic Analysis

To identify the causal agent, a single bacterial colony was added to a tube containing 20 µL of lysis buffer provided with the Mix MF848 kit (Mei5 Biotechnology Co., Beijing, China), three single colonies per isolate. Following the manufacturer’s instructions, the mixture was heated at 98 °C for 5 min and then centrifuged at 12,000 rpm for 2 min. PCR amplification was performed using 1 µL of the supernatant as template with the same kit. Multiple gene regions were amplified and sequenced [6]. The primer pairs listed in Table S1 were used to amplify partial sequences of the 16S rDNA, *gyrB*, *dnaK*, *efP*, and *atpD* gene sequences (Table S1). The PCR mixture and conditions for all gene regions followed established methods [6]. Amplification products were verified by 1% (*w*/*v*) agarose gel electrophoresis and sequenced by a commercial provider (Beijing Tsingke Biotech Co., Ltd., Beijing, China).

Sequence queries were conducted using the NCBI BLASTn (https://blast.ncbi.nlm.nih.gov/Blast.cgi; accessed on 1 April 2026) algorithm. Phylogenetic analyses incorporated additional sequences from NCBI (Table S2). Sequences for each locus were aligned with ClustalX1.83 [15], manually refined in MEGA7.0 [16], then concatenated in BioEdit7.0.9.0 [17]. Phylogenetic trees were reconstructed using neighbor-joining (NJ) and maximum likelihood (ML) methods with 1000 bootstrap replicates in MEGA7.0. The p-distance was used for NJ, and the Kimura 2-parameter model was used for ML.

### 2.5. Inoculum Preparation and Bacterial Growth Rate Determination

The inoculum was prepared by inoculating a single colony into 2 mL of KB broth followed by shaking at 180 rpm, 28 °C for 24 h. To determine the bacterial growth rate, 3 μL of the suspension was transferred into 3 mL of fresh KB broth and incubated for 36 h. Three replicate flasks were used, with measuring OD_600_ at 3 h intervals. At each time point, three 1 mL samples per flask were measured individually. The experiment was performed twice.

### 2.6. Effects of Culture Conditions on Bacterial Growth

To evaluate the cultural characterization of TCK-5, 3 μL of inoculum was inoculated into 3 mL of sterile liquid medium (controls received 3 μL of sterile medium), with cultures incubated at 28 °C and 180 rpm for 24 h unless otherwise specified, and growth was quantified by OD_600_. All experiments were performed twice with three replicates per treatment. Growth kinetics parameters were calculated based on OD_600_ measurements during the exponential phase. The specific growth rate (μ) was calculated using Formula (1); the doubling time (td) was calculated using Formula (2). The formulas are as follows:

(1) Specific growth rate (μ) = (ln(N_2_) − ln(N_1_))/(t_2_ − t_1_)

where N_1_ and N_2_ are OD_600_ values at time points t_1_ and t_2_ within the exponential phase.

(2) Doubling time (td) = ln(2)/μ

Culture media assays were conducted in NB, PDB, LB, KB, YEM and BPA media (formulations in Table S3). For all other culture condition assays, KB medium was used as the basal control. For light and temperature assays, cultures were grown under continuous light, continuous darkness, or a 12 h light/dark photoperiod; or at temperatures of 0, 5, 10, 15, 20, 25, 28, 30, 35, 40, or 45 °C. For pH and salinity assays, KB broth was adjusted to pH 3.0–12.0 using 0.1 M HCl or NaOH, or supplemented with 0.5–12.0% (*w*/*v*) NaCl prior to inoculation. Glycerol was replaced with an equal amount of sucrose, lactose, starch, mannitol, sorbitol, glucose, or maltose for carbon source testing; while proteose peptone was replaced with beef extract, yeast extract, lysine, glycine, tryptone, ammonium chloride, or peptone for nitrogen source testing.

### 2.7. Lethal Temperature Determination

To determine the lethal temperature for the isolates, aliquots (1 mL) of the inoculum were transferred to sterile 2 mL tubes and immersed in water baths at 46–55 °C (1 °C intervals) for 20 min. Subsequently, 10 uL of each heat-treated suspension was pipetted onto NA agar plates, air-dried, and incubated at 28 °C to assess survive. Each experiment was conducted twice with three replicates per temperature.

### 2.8. Bactericide Screening

The antibacterial activities of 11 chemical and 7 biological pesticides against the bacterial isolate were assessed using the filter paper disk agar diffusion method [18]. The bacterial suspension was prepared at OD_600_ of 0.5 and mixed thoroughly with cooled (~45 °C) NA medium at a 1:100 (*v*/*v*) ratio before pouring into 60 mm sterile Petri dishes. After solidification, 5 mm sterile filter paper discs immersed in each bactericide solution, respectively, were placed on the center of the plates. According to the preliminary tests, each bactericide was serially diluted with sterile water to obtain graded concentration series at serial dilutions (e.g., Zhongshengmycin at concentrations of 15, 30, 75, 150, and 300 mg/L; see Table S4 for complete concentration series for all bactericides), with sterile water as the negative control. All treatments were performed in triplicate. Plates were incubated at 28 °C for 48 h, after which inhibition zone diameters were measured using the cross-hatch method [19]. The inhibition rate was calculated for each concentration based on Formula (3). The effective concentration causing 50% inhibition of bacterial growth (EC_50_) value was determined via probit regression analysis of the percentage inhibition against the log10-transformed concentrations using SPSS 22.0 (IBM Corp., Armonk, NY, USA).

(3) Inhibition rate (%) = [(Dt − Dc)/Dt] × 100%

Dt denotes the diameter of inhibition zone in treatment group, and Dc indicates the diameter of inhibition zone in control group.

### 2.9. Data Analysis

Data were processed and analyzed using Microsoft Excel 2013 (Microsoft Corp., Redmond, WA, USA). Statistical analyses were performed with SPSS 22.0 (IBM Corp., Armonk, NY, USA). Prior to analysis, data were tested for normality using the Shapiro–Wilk test and for homogeneity of variance using Levene‘s test; all data met the assumptions of the tests (*p* > 0.05). Differences in the biological characteristics of the pathogen were analyzed by one-way analysis of variance (ANOVA), and multiple comparisons were conducted with Duncan’s multiple range test (DMRT) at a 95% confidence interval.

## 3. Results

### 3.1. Disease Symptoms

**Figure 1:**
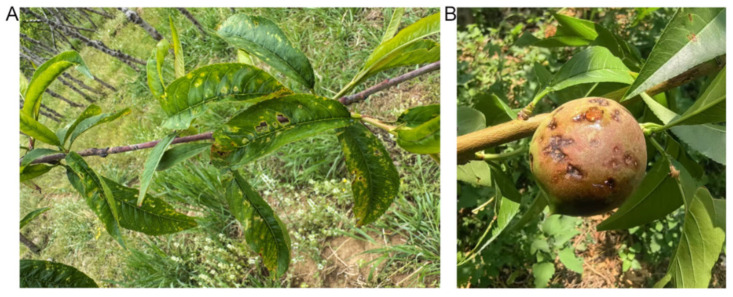
Symptoms of bacterial shot hole on peach leaves (**A**) and fruit (**B**).

During surveys of peach orchards in Changli County, severe disease symptoms were observed on both leaves and fruits in orchards aged 8 years. On leaves, symptoms initially appear as water-soaked spots along the veins and margins, progressing to dark necrotic lesions and ultimately resulting in shot hole symptoms (Figure 1A). On fruits, symptoms appear as sunken, brown lesions that may crack as the fruit expands (Figure 1B). The disease occurs throughout the growing season, affecting both fruit quality and tree health.

### 3.2. Pathogen Isolation and Pathogenicity Study

**Figure 2:**
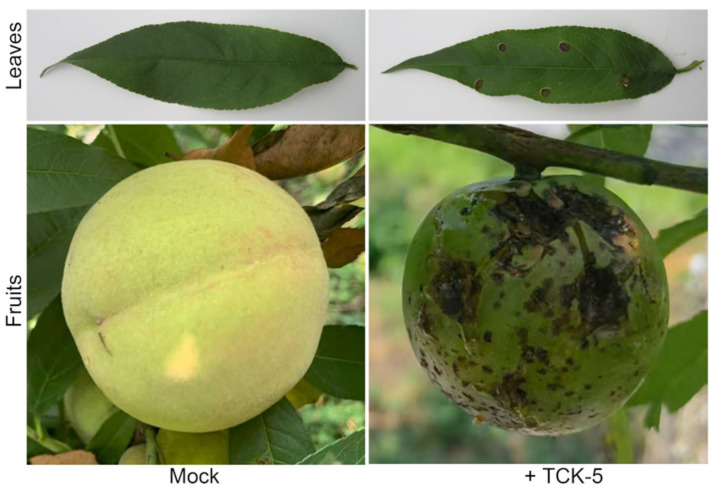
Pathogenicity of isolate TCK-5 to *P. persica* cv. Zhongtaohongyu. Field-grown peach leaves and fruits were inoculated with sterile distilled water (Mock) and with 0.05 OD_600_ of bacterial isolate (TCK-5), respectively. Inoculated tissues were maintained humidity with plastic bags for 48 h. Representative tissues were photographed at 14 d after inoculation. The experiment was repeated twice and similar results were observed.

A total of nine bacterial strains were isolated from diseased peach leaves exhibiting bacterial shot hole symptoms. To assess pathogenicity, each isolate was inoculated onto healthy peach leaves and fruits in the field. Only one isolate, TCK-5, formed dark brown shot hole lesions on leaves and sunken lesions on fruits, with the artificially infected incidence of 100% (20 leaves and 6 fruits per treatment). None of the control leaves and fruits showed any disease or symptoms (Figure 2). The pathogen was reisolated from the inoculated tissues after showing typical symptoms and confirmed to be identical to TCK-5, satisfying Koch’s postulates. These results demonstrate that strain TCK-5 is the causal agent of bacterial shot hole disease in peach.

### 3.3. Morphological and Biochemical Identification

**Figure 3:**
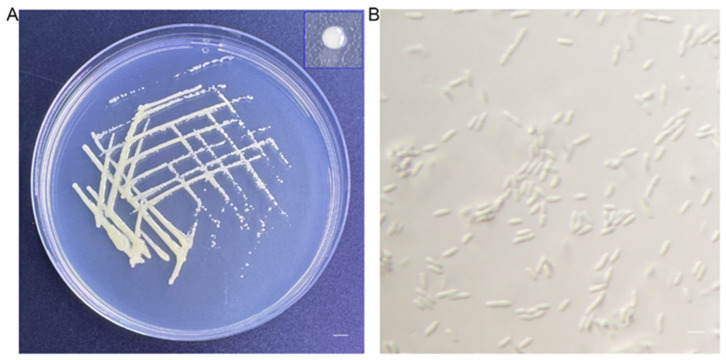
Morphological characters of pathogens isolated from disease peach. (**A**) Streak plate of bacteria isolate TCK-5 on NA for 48 h at 28 °C in the dark. The area within the blue box is magnified 8.5× in proportion. Scale bar = 3 mm; (**B**) microscopy (10 × 100) of TCK-5. Scale bars = 2 μm.

On NA agar plates, colonies of isolate TCK-5 were pale yellow, circular, convex, wet shining, and of a slimy mucoid consistency (Figure 3A). Bacterium of isolate TCK-5 was 0.5–1.1 μm × 1.5–2.3 μm, motile, straight rod, with bluntly rounded ends (Figure 3B). Gram staining showed that isolate TCK-5 appeared pinkish red, indicating a Gram-negative reaction (Table 1).

In biochemical tests, the isolate exhibited negative results for oxidase (no violet color), esculin hydrolysis (no blackening), and nitrate reduction (no red color). In contrast, positive reactions were observed for catalase (bubble produced), starch hydrolysis (clear halos produced around colonies), and H_2_S production (blackening of the medium). Additionally, gelatin liquefaction (medium liquefied) and citrate utilization (color changed from green to blue) were also positive (Table 1). These morphological and biochemical characteristics corresponded to those of typical *Xanthomonas* spp., providing critical evidence for taxonomic identification of isolate TCK-5.

### 3.4. Molecular Identification and Phylogenetic Analysis

**Figure 4:**
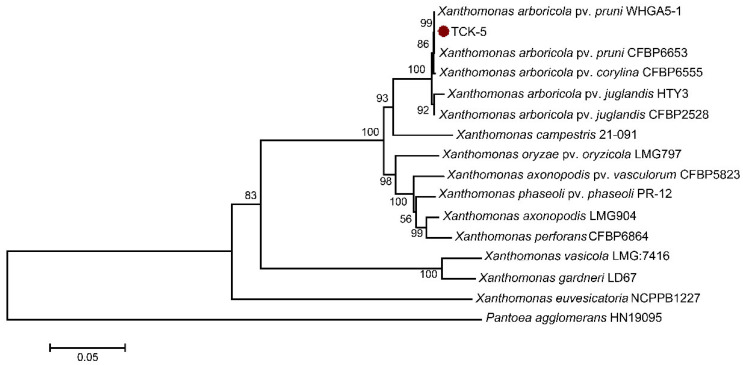
Phylogenetic analysis of peach bacterial shot hole pathogen based on the NJ method. Multi-locus phylogeny of TCK-5 and related *Xanthomonas* species inferred from combined *gyrB*, *dnaK*, *efP*, and *atpD* sequences. Aligned gene boundaries spanned 1–796 bp, 797–1429 bp, 1430–1768 bp, and 1769–2512 bp, respectively. Scale bar indicates 0.05 substitutions per nucleotide position. Bootstrap support values (1000 repetitions) are indicated at nodes. The isolate analyzed in this study (TCK-5) is emphasized with red dots. *Pantoea agglomerans* strain HN19095 was used as the outgroup.

Molecular characterization was carried out based on 16S rDNA, *gyrB*, *dnaK*, *efP*, and *atpD* genes. In all cases, single bands of the expected sizes (16S rRNA: ~1500 bp; gyrB: ~800 bp; dnaK: ~750 bp; efP: ~350 bp; atpD: ~750 bp) were observed, with no evidence of non-specific amplification or primer-dimer formation. Representative bands were excised and purified for bidirectional sequencing. The five gene sequences of TCK-5 isolate were submitted to GenBank as PZ287798, and PZ291676-PZ291679, respectively. BLAST searches of the obtained sequences revealed 100% (1423 bp/1423 bp, 818 bp/818 bp, 761 bp/761 bp, 339/339, and 750 bp/750 bp, respectively) homology on DNA base-pair matching with those of *Xanthomonas arboricola* pv. *pruni* (strain T1) in GenBank. Multi-locus phylogenetic analyses of the combined *gyrB*-*dnaK*-*efP*-*atpD* loci sequence alignment using the NJ method revealed that TCK-5 clustered with reference strains of *X. arboricola* pv. *pruni* (including WHGA5-1 and CFBP6653) with 99% bootstrap-support (Figure 4). The ML tree showed a topology consistent with the NJ tree, confirming its identification as *X. arboricola* pv. *pruni*.

### 3.5. Optimization for TCK-5 Growth In Vitro

**Figure 5:**
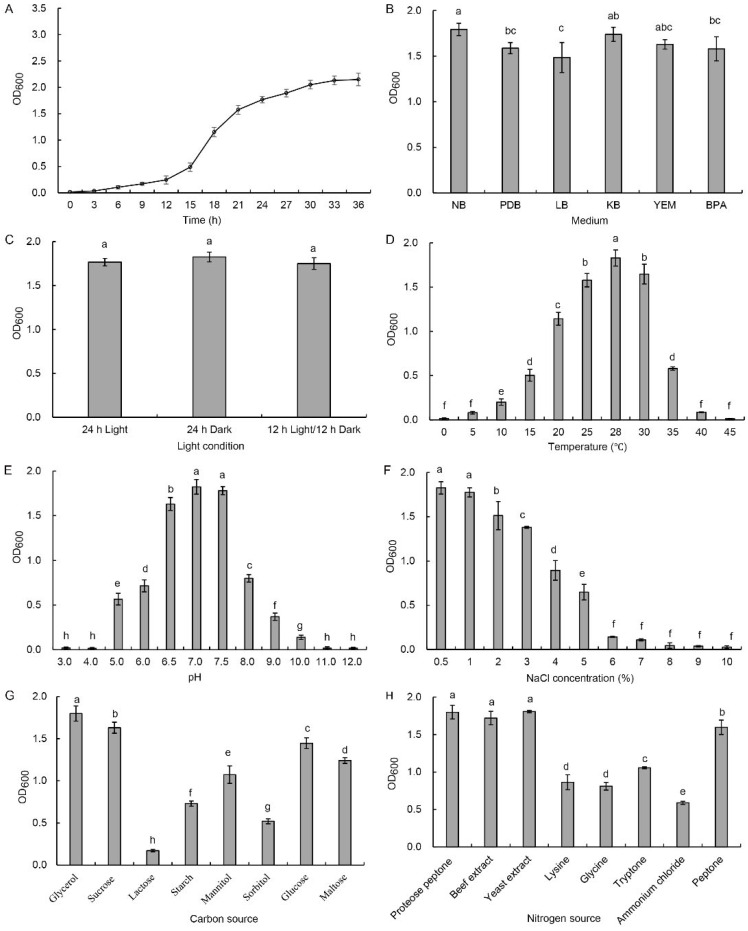
Effects of various culture conditions on TCK-5 growth. (**A**) Growth curve of TCK-5 in KB for 36 h. (**B**–**H**) Effect of different culture media (**B**), light conditions (**C**), temperature (**D**), pH (**E**), NaCl concentration (**F**), carbon sources (**G**), and nitrogen sources (**H**) on TCK-5 growth for 24 h. Data are presented as mean ± standard deviation (SD) of three replicates. The vertical bars indicate SD. Different lowercase letters above bars indicate significant differences (*p* < 0.05) according to one-way ANOVA followed by Duncan’s multiple range test.

Previous studies have demonstrated that multiple factors, including incubation time, culture conditions, and nutrients, impact bacterial growth [20]. To establish the growth kinetics of isolate TCK-5, we monitored its growth over time in liquid culture by measuring OD_600_. The growth curve showed an initial lag phase of approximately 15 h, followed by exponential growth until 24 h, after which the culture entered stationary phase with OD_600_ stabilizing at 1.8–2.2 (Figure 5A). During the exponential phase (from 15 h to 24 h), the specific growth rate (μ) was calculated to be 0.143 h^−1^, with a doubling time (td) of 4.8 h, indicating that TCK-5 exhibits relatively moderate growth kinetics under the tested conditions. We then evaluated the effect of different media on bacterial proliferation. Among six tested media, bacterial growth was significantly higher in NB, KB, YEM and BPA than in PDB or LB, with optimal bacterial density (OD_600_ ~ 1.8) in both KB and NB medium (Figure 5B). Thus, KB medium with a 24 h incubation was selected for subsequent assays.

To investigate whether light, temperature, pH, and NaCl concentration affect the growth of TCK-5, we monitored bacterial proliferation by measuring OD_600_. The results showed that light conditions (24 h light, 24 h dark, or 12 h light/12 h dark) did not significantly affect TCK-5 growth (Figure 5C). In contrast, temperature had a marked influence, with the optimal growth occurring at 28 °C, whereas growth was negligible below 5 °C and above 40 °C (Figure 5D). In addition, TCK-5 exhibited optimal growth at pH 7.0–7.5 and NaCl concentrations of 0.5–1.0%, with OD_600_ values reaching approximately 1.7–1.8, while growth was strongly inhibited at pH ≤ 6 or ≥9, or at NaCl concentrations above 3.0% (Figure 5E,F).

For carbon and nitrogen source utilization, glycerol supported the highest growth, followed by sucrose, glucose and maltose, which was 7–10-fold higher than that of the least utilized lactose (OD_600_ of 0.17). Intermediate growth was observed for mannitol, starch, and sorbitol (3–6-fold higher than lactose). Proteose peptone, beef extract, and yeast extract were the most favorable nitrogen sources (OD_600_ = 1.7–1.8), followed by peptone (OD_600_ = 1.6), while ammonium chloride was the least effective (Figure 5H).

As shown in Table 2, isolate TCK-5 retained viability at temperatures ranging from 46 to 50 °C, with visible colonies recovered on KB plates (+). At 51 °C and above, no viable colonies were detected (−), indicating that the lethal temperature for TCK-5 was 51 °C. These results demonstrate that TCK-5 exhibits moderate thermotolerance, surviving at temperatures up to 50 °C, with complete inhibition occurring at 51 °C.

### 3.6. Indoor Virulence Determination of Bactericides Against Isolate TCK-5

Among the 18 tested bactericides, biological bactericides generally exhibited greater efficacy than chemical ones (Table 3). The 0.3% Tetramycin AS showed the strongest inhibitory activity against strain TCK-5, with the lowest EC_50_ value of 0.1051 mg/L, followed by 3% Zhongshengmycin SL (EC_50_ = 2.9252 mg/L). The remaining biological bactericides displayed moderate inhibitory activity, with EC_50_ values below 60 mg/L. Only one chemical bactericide, 20% Bronopol SC, displayed relatively higher activity (EC_50_ = 5.7931 mg/L). In contrast, several chemical bactericides, such as 40% Tebuconazole·Zinc Thiazole SC, 52% Copper Oxychloride·Zineb WP, and 30% Thiodiazole Copper SC, were the least effective, with EC_50_ values exceeding 28,000 mg/L. These findings suggest that biological bactericides, particularly Tetramycin and Zhongshengmycin, possess superior inhibitory activity against strain TCK-5 under indoor conditions.

## 4. Discussion

Accurate pathogen identification is the primary step in controlling plant diseases. Combining genotypic and phenotypic data has become a prevailing trend in precise identification. In this study, the causal agent of peach bacterial shot hole was identified as *Xanthomonas arboricola* pv. *pruni* based on morphological observation, physiological and biochemical characterization and multi-locus phylogenetic analysis. Our results are consistent with a recent study, which reported that the strains H-1, L-3 and Q-7 of *X*. *arboricola* pv. *pruni* with similar phenotypic characteristics were the causal agent of peach bacterial leaf spot in Shenzhou and Shunping in Hebei Province [3]. This integrated approach significantly enhanced taxonomic resolution, enabling reliable discrimination from closely related pathovars such as *X. arboricola* pv. *juglandis* or *X. arboricola* pv. *corylina* [21].

Previous studies have demonstrated that *X. arboricola* pv. *pruni* is the dominant pathogen worldwide, posing a serious threat to other economical stone fruits, e.g., plum, apricot and cherry [18]. Subsequent investigations in Henan, Liaoning and other production regions in China have successively identified pathogens including *Pantoea agglomerans*, *P. ananatis* and *Bacillus cereus*, suggesting the geographical diversity of causal agents associated with peach bacterial shot hole [4,6]. In contrast, our study confirmed that *X. arboricola* was the sole pathogen in Hebei, with no *Pantoea* spp. or *Bacillus* spp. detected, which is highly consistent with the previous findings [7]. Presumably, the unique temperate continental climate, soil physicochemical properties and genetic background of dominant peach cultivars in Hebei may restrict the colonization and pathogenicity of other bacteria, providing a critical pathological basis for formulating region-specific control strategies.

The biological characteristics of pathogens determine their environmental adaptability and disease epidemic patterns, enabling accurate prediction of disease occurrence risks and optimization of control timing [22]. Our cultural optimization experiments revealed that pathogenic bacteria (TCK-5) has strong environmental adaptability. Its growth was dependent on temperature, with optimal growth at 28 °C, which is consistent with the optimal growth temperature (27–29 °C) reported for various *Xanthomonas* species [23,24,25]. Moreover, the 25–30 °C range (optimum 28.9 °C) is optimal for disease development in *X. arboricola* pv. *pruni*, yielding the shortest incubation periods [23]. This aligns with the peak disease incidence typically observed in stone fruits during warm, humid growing seasons [23]. More significantly, we determined the lethal temperature for TCK-5 to be 51 °C, which is notably higher than the 47 °C reported for other *Xanthomonas* species (e.g., *X. fragariae* [25]), suggesting that TCK-5 possesses greater thermotolerance compared to those *Xanthomonas*. This finding implies that hot water treatment (above 50 °C) of pruning tools, dormant nursery stock, or even seeds for a defined period could be an effective strategy to prevent pathogen introduction and spread, a method successfully used against other bacterial pathogens [26]. Furthermore, the pathogen’s strict requirement for alkaline pH (7.0–7.5) and low salinity (0.5–1.0% NaCl) revealed that deviations (pH of ≤6 or ≥9, NaCl > 3%) severely inhibit growth. The salinity tolerance range is generally consistent with that of other *Xanthomonas* species [25], suggesting potential strategies for manipulating orchard environment (e.g., soil pH adjustment) or developing suppressive formulations.

The most impactful findings for disease management arise from the in vitro bactericide sensitivity assays. The results revealed that biological bactericides, specifically 0.3% Tetramycin AS and 3% Zhongshengmycin SL, exhibited orders of magnitude greater inhibitory activity against TCK-5 compared to most chemical agents. This finding is consistent with previous reports on almond bacterial shot hole disease caused by *Pseudomonas* spp., where the same biological bactericides also showed superior performance [27]. Their exceptional efficacy stems from the multi-target mechanisms of polyene macrolide antibiotic Tetramycin and precise protein synthesis inhibition of nucleoside antibiotic Zhongshengmycin, both derived from *Streptomyces* spp. [18,28]. In contrast, the isolate TCK-5 was insensitive to copper-based pesticides, such as 52% Copper Oxychloride·Zineb WP and 30% Thiodiazole Copper SC. Cupric pesticides have been the mainstay for bacterial disease control for decades, and the widespread emergence of copper-resistant strains has gained global attention, suggesting that the continued application of such products in this region is likely ineffective and may result in higher resistance levels [7]. Several other chemical bactericides (e.g., 40% Tebuconazole·Zinc Thiazole SC) exhibited very low inhibitory activity under the tested conditions. Nevertheless, the extremely high EC_50_ values (>28,000 mg/L) should be interpreted cautiously, as precipitation or formulation incompatibility cannot be excluded, although we did not observe visible precipitation in our preliminary tests. In addition, in this study, EC_50_ values were used as the criterion for selecting bactericides against *X. arboricola* pv. *pruni*, while the concentrations used in field applications are usually higher than those in vitro, and field control efficacy also varies greatly across regions and plant tissues [7]. These differences are due to environment and physiological factors, such as temperature, rain wash-off, active ingredient degradation, tissue structures and surface adherence [29,30]. Furthermore, previous studies have confirmed that Tetramycin exhibits excellent field control efficacy of 82.5% against *X. arboricola* pv. *pruni* under [3], which is consistent with our in vitro results showing strong inhibitory activity of Tetramycin against isolate TCK-5. The strong agreement between our in vitro data and previous field validation further supports the conclusion that Tetramycin can serve as an effective alternative to copper-based bactericides for the management of peach bacterial shot hole.

However, certain limitations should be noted, as these in vitro results were obtained under controlled conditions that do not fully capture the complexity of field environment. Factors such as fluctuations in temperature and humidity, UV degradation, and plant systemic activity can significantly influence the efficacy of bactericides [7,31]. Therefore, field trials are urgently needed to validate the performance of Tetramycin, Zhongshengmycin, and Bronopol before definitive recommendations can be made. Specifically, we propose that future field trials should apply these bactericides twice with 7–10 day spray intervals during the early disease stage; both preventive and curative treatment schedules should be evaluated; additionally, assessments of rainfastness and residual activity are recommended to guide practical application. Furthermore, the observed high copper tolerance and the lethal temperature identified in this study raise important biological questions. Future research could focus on whole-genome sequencing of TCK-5 to uncover the genetic determinants underlying its copper resistance, thermotolerance, and virulence. In addition, although Koch’s postulates were fulfilled, a quantitative disease index was not calculated in this study. A limitation of the pathogenicity assay is the absence of a standardized disease severity grading scale and statistical analysis; future studies should incorporate these elements to improve scientific rigor.

## 5. Conclusions

In this study, *Xanthomonas arboricola* pv. *pruni* was identified as the causal agent of peach bacterial shot hole disease in Hebei Province, China. Biological characterization established optimal growth conditions (28 °C, pH 7.0–7.5, 0.5–1.0% NaCl, glycerol, proteose peptone, beef and yeast extract as carbon and nitrogen source) and lethal temperature of 51 °C. Notably, biological bactericides, particularly Tetramycin and Zhongshengmycin, exhibited superior efficacy against TCK-5 in vitro. These findings provide the comprehensive characterization of *X. arboricola* pv. *pruni* and offer a scientific basis for improving disease management strategies.

## Figures and Tables

**Table 1:** Biochemical characteristics of TCK-5.

Test	Observation	Results
Gram staining	Cells pinkish red	−
Oxidase	No violet color	−
Catalase	Bubbles produced	+
Starch hydrolase	Clear halo around colonies	+
H_2_S production	Medium turned black	+
Gelatin hydrolysis	Liquefaction	+
Esculin hydrolysis	No black color	−
Nitrate reduction	No red color	−
Citrate utilization	Color changed from green to blue	+

Note: “+” and “−” represent positive and negative reactions, respectively.

**Table 2:** The lethal temperature of TCK-5.

	Temperature (°C)									
	46	47	48	49	50	51	52	53	54	55
Appearance	+	+	+	+	+	−	−	−	−	−

“+” means that the visible colonies appeared on the KB plates; “−” means that no visible colonies appeared on the KB plates.

**Table 3:** Indoor virulence determination of eighteen bactericides against strain TCK-5.

Bactericides	Toxicity Regression Equation	EC_50_ (mg/L)	Coefficient (R)
0.3% Tetramycin AS ^a^	y = 0.3295x + 5.3224	0.1051	0.9918
3% Zhongshengmycin SL ^a^	y = 0.2618x + 4.8780	2.9252	0.9755
20% Bronopol SC ^b^	y = 0.9611x + 4.7684	5.7931	0.9611
2% Zhongshengcin·Tetracycline SC ^a^	y = 0.5173x − 4.2328	30.4126	0.9861
1 × 10^9^ spores/g Bacillus amyloliquefaciens B7900 WP ^a^	y = 4.0932x − 1.3362	35.3182	0.8498
8 × 10^9^ spores/mL Bacillus licheniformis AS ^a^	y = 0.6306x + 3.9168	52.2061	0.9080
1 × 10^9^ CFU/g Paenibacillus polymyxa WP ^a^	y = 5.0479x − 3.8911	57.7241	0.9509
35% Quinolinone·Tetramycin SC ^b^	y = 0.4283x + 4.2251	64.4231	0.9617
60% Pyraclostrobin·Metiram WG ^b^	y = 0.2150x + 4.6022	70.8361	0.8891
3% Benziothiazolinone ME ^b^	y = 0.5592x + 3.7478	173.5425	0.9912
2% Kasugamycin AS ^a^	y = 4.7554x − 9.5458	1145.0433	0.8990
50% Chloroisobromine Cyanurate SP ^b^	y = 0.8207x + 2.1963	2607.1132	0.9495
40% Zinc Thiazole SC ^b^	y = 0.4465x + 3.3193	5807.5958	0.8918
80% Mancozeb WP ^b^	y = 0.4139x + 3.3516	9614.5734	0.9724
45% Kasugamycin·Quinolinone SC ^b^	y = 2.7073x − 6.1236	12,845.5096	0.9222
40% Tebuconazole·Zinc Thiazole SC ^b^	y = 2.8161x − 7.5459	28,511.7524	0.8114
52% Copper Oxychloride·Zineb WP ^b^	y = 1.8854x − 3.4000	28,531.4094	0.8028
30% Thiodiazole Copper SC ^b^	y = 4.0167x − 13.0720	31,563.7135	0.9627

SL, soluble concentrate; WP, wettable powder; ME, microemulsion; SC, suspension concentrate; AS, aqueous solution; WG, water dispersible granule; SP, soluble powder. ^a^ Biological bactericide, ^b^ chemical bactericide.

## Data Availability

The original contributions presented in this study are included in the article and Supplementary Material. Further inquiries can be directed to the corresponding authors.

## Supplementary Materials

The following supporting information can be downloaded at: https://www.mdpi.com/article/10.3390/microorganisms14061179/s1, Table S1: Gene regions and primers used in this study; Table S2: The bacterial isolates information used for the phylogenetic analysis in this study; Table S3: The basically culture medium formulations; Table S4: Bactericides used in this study.
