# Supplementary material for: Identification, Biology, and Bactericide Control of Peach Bacterial Shot Hole in Hebei Province, China
Source: Microorganisms. 2026 May 23;14(6):1179. doi: 10.3390/microorganisms14061179 (PMC13304292; doi:10.3390/microorganisms14061179)
Supplement: Supplementary file 1 [file microorganisms-14-01179-s001.zip › microorganisms-4295661-supplementary.pdf]

**Table S1.** Gene regions and primers used in this study.

| Gene region | Primer pairs | Sequences (5′-3′) | Amplicon size (bp) | Reference |
|---|---|---|---|---|
| 16S rDNA | 27-F | AGAGTTTGATCCTGGCTCAG | ~1,500 | [6] |
| | 1492-R | TACGGTTACCTTGTTACGACT | | |
| *gyrB* | gyrB-Fb | GCCGAGGTGATCCTCACCGT | ~800 | [7] |
| | gyrB-Rb | GGCCGAGCCACCTGCCGAGT | | |
| *dnaK* | dnaK-Fc | TGGGCAAGATCATTGGTATT | ~750 | |
| | dnaK-Rc | ACCTTCGGCATACGGGTCTG | | |
| *efP* | efp-Fc | GTCAAGAACGGCATGAAGA | ~350 | |
| | efp-Rc | TCGTCCTGGTTGACGAAC | | |
| *atpD* | atpD-Fc | GGGCAAGATCGTTCAGAT | ~750 | |
| | atpD-Rc | GCTCTTGGTCGAGGTGAT | | |

Note: 16S rDNA, 16S ribosomal DNA; *gyrB*, DNA gyrase subunit B; *dnaK*, the chaperone protein dnaK; *efP*, the elongation factor P; *atpD*, the ATP synthase subunit beta gene.

**Table S2.** The bacterial isolates information used for the plylogenetic analysis in this study

| | | GenBank Accession Number | | | |
|---|---|---|---|---|---|
| Species | Isolates | gyrB | dnaK | efP | atpD |
| *Xanthomonas arboricola pv. pruni* | TCK-5 | PZ291676 | PZ291677 | PZ291678 | PZ291679 |
| | CFBP6653 | KF904908 | KF904504 | KF904605 | KF904403 |
| | WHGA 5-1 | MW504073 | MW504094 | MW504115 | MW504136 |
| *X. arboricola pv. juglandis* | CFBP2528 | KF904869 | KF904465 | KF904566 | KF904364 |
| | HTY3 | MW504090 | MW504111 | MW504132 | MW504153 |
| *X. arboricola pv. corylina* | CFBP6555 | KT874028 | KT874284 | KT874220 | KT874348 |
| *X. axonopodis pv. vasculorum* | CFBP5823 | HQ591263 | HQ590739 | HQ590870 | HQ590608 |
| *X. perforans* | CFBP6864 | HQ591287 | HQ590763 | HQ590894 | HQ590632 |
| *X. oryzae pv. oryzicola* | LMG797 | JX886972 | HM569029 | JX886892 | JX682665 |
| *X. phaseoli pv. phaseoli* | PR-12 | ON416689 | ON416709 | ON416727 | ON416671 |
| *X. euvesicatoria* | NCPPB1227 | GU322515 | GU322685 | GU322600 | GU322770 |
| *X. vasicola* | LMG:7416 | HM569207 | HM569040 | HM569123 | HM568956 |
| *X. axonopodis* | LMG904 | KJ491532 | EU015264 | FJ376347 | EU015172 |
| *X. gardneri* | LD67 | GU322535 | GU322705 | GU322620 | GU322790 |
| *X. campestris* | 21-091 | OR493151 | OR493200 | OR493188 | OR493212 |
| *Pantoea agglomerans* | HN19095 | MW167146 | - | - | - |

**Table S3.** The basically culture medium formulations

| Medium | Formulations (per liter) | Reference |
|---|---|---|
| NA | Beef extract: 5 g; Peptone: 5 g; NaCl: 5 g; Agar: 15 g; the pH was adjusted to 7.2. | [27] |
| NB | Beef extract: 5 g; Peptone: 5 g; NaCl: 5 g; the pH was adjusted to 7.2. | |
| PDB | Potato infusion: 200 g; Glucose: 20 g; the pH was adjusted to 7.2. | |
| KB | Proteose peptone: 20 g; $K_2HPO_4$:1.5 g; $MgSO_4{\cdot}7H_2O$: 0.4 g; Glycerol: 10 mL; the pH was adjusted to 7.2. | |
| LB | Peptone: 10 g; Yeast extract: 5 g; NaCl: 10 g; the pH was adjusted to 7.2. | |
| YEM | Mannitol: 10 g; Yeast extract: 3 g; $K_2HPO_4$: 0.5 g; NaCl: 0.2 g; $MgSO_4$: 0.2 g; the pH was adjusted to 7.2. | |
| BPA | Peptone: 5 g; Sucrose: 10 g; Beef extract : 3 g; Yeast extract:1 g; the pH was adjusted to 7.2. | |

**Table S4**. Bactericides used in this study

| Bactericides | Manufacturer | Category | Amount of active ingredient/mg·L$^{-1}$ |
|---|---|---|---|
| 80% Mancozeb WP | Jiangsu Yunfan Chemical Co. Ltd. | Chemical bactericide | 8000, 16000, 40000, 64000, 80000 |
| 3% Benziothiazolinone ME | ADAMA Huifeng (jiangsu) Co. Ltd. | Chemical bactericide | 300, 600, 1200, 1500, 3000 |
| 45% Kasugamycin·Quinolinone SC | Xingnong Chemical (China) Co.,Ltd. | Chemical bactericide | 450, 2250, 9000, 22500, 45000 |
| 20% Bronopol SC | Shaanxi Thompson Biological Technology Co., Ltd. | Chemical bactericide | 1000, 2000, 10000, 20000, 40000 |
| 40% Zinc Thiazole SC | Zhejiang Xinnong Chemical Co., Ltd. | Chemical bactericide | 4000, 20000, 40000, 80000, 200000 |
| 40% Tebuconazole·Zinc Thiazole SC | Zhejiang Xinnong Chemical Co., Ltd. | Chemical bactericide | 2000, 4000, 20000, 40000, 80000 |
| 35% Quinolinone·Tetramycin SC | Qingdao Aodesi Biological Technology Co., Ltd. | Chemical bactericide | 1750, 3500, 17500, 35000, 70000 |
| 60% Pyraclostrobin·Metiram WG | Foshan Yinghui Crop Science Co., Ltd. | Chemical bactericide | 3000, 6000, 30000, 60000, 120000 |
| 50% Chloroisobromine Cyanurate SP | Nanjing Nannong Pesticide Technology Development Co., Ltd. | Chemical bactericide | 5000, 25000, 50000, 100000, 200000 |
| 30% Thiodiazole Copper SC | Zhejiang Dongfeng Chemical Co., Ltd. | Chemical bactericide | 1500, 3000, 15000, 30000, 60000 |
| 52% Copper Oxychloride·Zineb WP | Shaanxi Sangear Road Biological Sciences Co., Ltd. | Chemical bactericide | 2600, 5200, 26000, 52000, 104000 |
| 0.3% Tetramycin AS | Liaoning Wkioc Bioengineering Co. Ltd. | Biological bactericide | 7.5, 15, 30, 150, 300 |
| 2% Kasugamycin AS | **S**handong Qingdao Glis Pharmaceutical Co., Ltd. | Biological bactericide | 100, 200, 400, 1000, 2000 |
| 2% Zhongshengcin·Tetracycline SC | Hailir Pharmaceutical Group Co., Ltd. | Biological bactericide | 20, 200, 1000, 2000, 4000 |
| $8\times10^9$ spores/mL *Bacillus licheniformis* AS | Guangxi Golden Swallow Biochemistry Co., Ltd. | Biological bactericide | 32, 160, 320, 640, 3200 |
| $1\times10^9$ spores/g *Bacillus amyloliquefaciens* B7900 WP | Shaanxi Xiannong Biological Technology Co., Ltd. | Biological bactericide | 5, 10, 20, 40, 80 |
| $1\times10^9$ CFU/g *Paenibacillus polymyxa* WP | Guangdong Goodefon Biological Technology Co., Ltd. | Biological bactericide | 5, 10, 20, 40, 80 |
| 3% Zhongshengmycin SL | Fujian Kaili Biological Products Co., Ltd. | Biological bactericide | 15, 30, 75, 150, 300 |
